# Regular Meeting of the Atlanta Society of Medicine

**Published:** 1895-03

**Authors:** W. L. Champion

**Affiliations:** 301½ Marietta Street


### Society Reports.

---

### REGULAR MEETING OF THE ATLANTA SOCIETY OF MEDICINE.

February 19, 1895.

The meeting was called to order by the President, Dr. C. D. Hurt.

Dr. J. W. Duncan read a paper on Coal-Tar Derivatives and their uses, published in this issue.

Dr. L. H. Jones opened the discussion by saying that he desired to call attention to antikamnia; that he never prescribed this and thought it ought not to be prescribed, as it was sold as a patent medicine, and prescribing it encouraged the sale of such medicines. However, it could be written in prescriptions without encouraging its sale as a patent medicine. Dr. Jones stated that he had had a reasonable amount of experience with the coal-tar derivatives, but the ones he used most were phenacetin, antipyrin and acetanelid. Phenacetin was a reliable antipyretic, but not as prompt as antipyrin for that purpose. For light pains it was superior to opium, etc., as always obtained desired results and no unpleasant effects. Always used it for neuralgia in preference to opium, although used the opium in all cases of deep-seated pains. In cases of fever where any three were used to reduce heat they were very reliable, but if used in large doses would produce a profuse perspiration and likely to cause collapse of patient, but had never seen this effect when used in small doses. However, had seen large doses used without any bad effect. Once gave a man antipyrin for neuralgia. Gave him 60 grains; 10 grains to a dose to be taken every three hours. The man left his office in the morning at 10 o'clock and returned in the evening without any medicine or sickness. He stated that he took one dose when he obtained the medicine and as he got all right, he took the remainder of it so as to make sure. The man did not suffer any bad effects. Dr. Jones stated that he himself had often taken 20 grain doses for headache without having any bad effects, but did not prescribe this amount. In relieving pain the antipyrin was superior to the phenacetin, and in cases of fever had seen the antipyrin fail where the phenacetin had given good results. Has used them with good results in cases of rheumatism and had never seen any serious unpleasant effects from any of them; in one case where antipyrin was used, it caused a slight eruption. In cases of children had used all three, and the phenacetin had given especially good results in relieving pain, but the antipyrin was the most soluble and for that reason favored prescribing it. It has a peculiar taste but does not remain long and is not disagreeable, and acts on the sensory nerves more than on the motor nerves. All three increase the tendency to take cold and for that reason should be taken when the person can stay in-doors. Had used salol some but without any especial good results.

DR. F. B. MCRAE stated that he was fully convinced of the good of the coal-tar derivatives; that he gave them in place of opiates, and almost invariably obtained the desired results. He was not attached more to one than to another, but had used a number of them and not much difference in them, simply a matter of adaptability of each one to each case, and all very satisfactory. Considered them superior to opiates and therefore used them instead.

DR. J. B. BAIRD was opposed to antikamnia and never prescribed it, as he understood it to be a composition of drugs. The ones he usually employed were antipyrin and phenacetin, and also antifebrin, but that he preferred the phenacetin to either of the others, as it accomplished the desired results without any unpleasant effects. They were excellent to reduce the temperature, but that he thought too much importance was attached to the simple existence of heat and the removing of it and not enough attention paid to its cause. In a number of cases it was a very good thing to use these heat reducers, as they rendered the patient comfortable, and were beneficial in that respect, but there are many conditions where their use is objectionable as in many diseases where the temperature runs high they should be used with great caution.

DR. L. B. GRANDY stated that in 1894 there were nearly two-hundred new preparations and most of them were coal-tar derivatives, but that he considered it safe to say that not more than one-tenth possess really valuable properties as curative agents, or properties that are claimed for them. That we had to depend too much upon what the chemists say and that laboratory experiments differ from bedside experience and it is best to rely upon fixed points than upon everything that chemists bring. The great majority of preparations will be found to have no advantage, or none which are not supplied by what has been used previously. Still there are some that have been brought out and especially three of the coal-tar derivatives. These are excellent to reduce heat, but indications are that they have not been used so much to reduce heat and to prevent its rising, and a rise of two or three degrees is not necessarily injurious to the system, and where it is necessary to reduce the temperature it is often better to use baths or something else. Salol has very valuable properties; used principally for diarrhœas of different kinds, and mostly with children. Can be controlled in children by small doses, probably two or three grains. Sulphonal consider reliable and safe. It rarely fails to produce sleep, its action being slow and sure.

DR. R. R. KIME was opposed to the too free use of the coal-tar derivatives in reducing heat in fevers, as in the majority of instances it does not eliminate the cause of the disease and in many cases damage and injury is done, as they destroy the known methods of ascertaining the condition of the patient, such as by temperature, pulse, etc.

DR. V. O. HARDON stated that of the coal-tar derivatives he used mostly phenacetin, antipyrin, antifebrin, and Sulphonal. Used some of the others, but very little. Has used all of the first three mentioned but as a result of experience, now uses the phenacetin almost entirely. Does not use much antipyretics for the reason stated by Dr. Kime, that it destroys the guide to find the cause of the trouble, by reducing temperature, etc. For neuralgia use phenacetin combined with powdered camphor to reduce the pain, as its energetic effect is increased. Use sulphonal almost entirely to produce sleep, as it rarely fails.

DR. T. D. LOVE stated that he had used several of the coal-tar derivatives quite extensively, but had found more benefit from antikamnia and salol. Had used salol in bowel troubles, dysentery, etc., and acts well, and used antipyrin in neuralgic conditions extensively.

DR. J. S. TODD stated that when the coal tar derivatives were first brought out, the old physicians used them extensively, as it was gratifying to them to be able to reduce the temperature so easily, and it was shown that the mortality in typhoid fevers increased thirty-five to forty per cent. Many people died, whose temperature did not rise above 100. All of these remedies are depressing, and any remedy which given to a patient in typhoid state takes away vitality is injurious. Glad that they were discovered, but have done such great damage in typhoid cases. They are good substitutes for opium and are invaluable in all states of inflammation. In a number of cases it is best not to give them, except in conjunction with some stimulant, such as camphor, etc. Do not prescribe them to children, except antipyrine, on account of want of solubility. Used icthyol for erysipelas, also for gonorrhœa twenty-five per cent. of water. Sulphonal not at all satisfactory.

W. L. CHAMPION, M. D.,

301½ *Marietta Street.* Secretary.

